# Supplementary material for: Retinal Ganglion Cell Survival and Axon Regeneration after Optic Nerve Transection is Driven by Cellular Intravitreal Sciatic Nerve Grafts
Source: Cells. 2020 May 27;9(6):1335. doi: 10.3390/cells9061335 (PMC7349876; doi:10.3390/cells9061335)
Supplement: Supplementary file 1 [file cells-09-01335-s001.pdf]

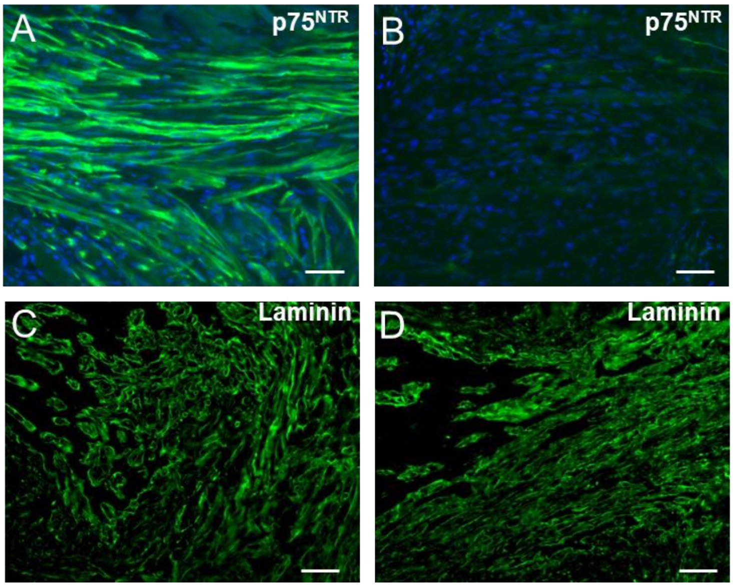

Figure S1. Schwann cells are absent from SN after freeze-thawing but basal lamina tubes remain intact. (**A**) p75^{NTR+} (green) with DAPI nuclear stain (blue) was used as a marker to detect Schwann cells (green) in fresh CSN, but not in freeze-thawed ASN grafts. (**B**) Laminin^{+} basal lamina tubes (green) were present in both (**C**) CSN and (**D**) ASN grafts. Scale bars for A–D = 50 μm.

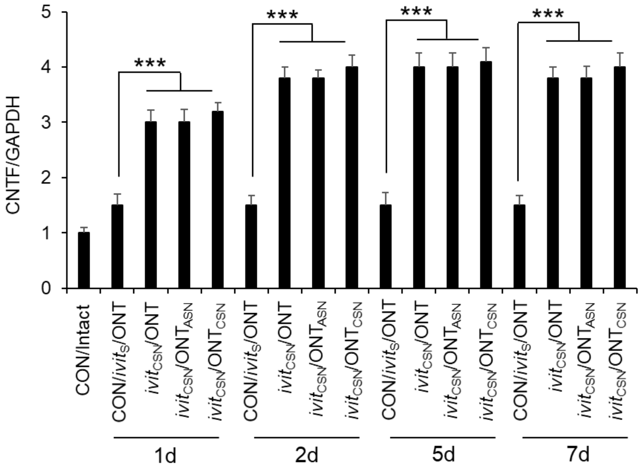

Figure S2. Relative mRNA levels of ciliary neurotrophic factor (CNTF) at different time points after grafting. ***$P < 0.0001$, ANOVA with Dunnett's *post hoc* test. GAPDH, Glyceraldehyde-3-phosphate dehydrogenase (housekeeping gene).

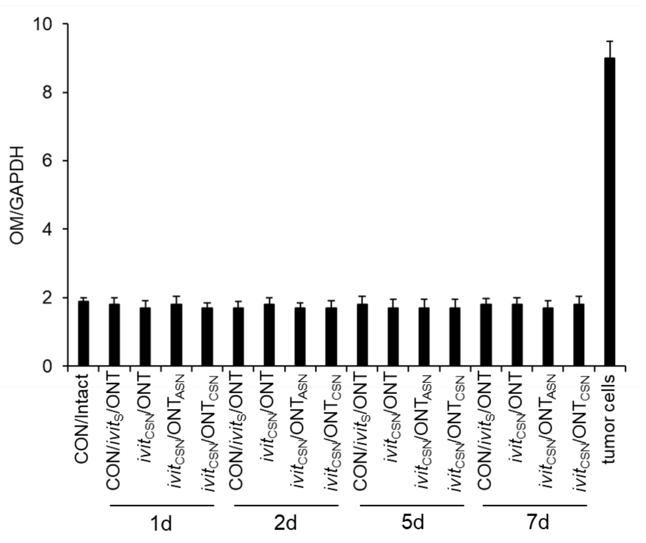

Figure S3. Relative levels of oncomodulin (OM) mRNA at different time-points after grafting. Tumor cells are used as a positive control. No significant difference in oncomodulin levels between experimental groups. GAPDH, Glyceraldehyde-3-phosphate dehydrogenase (housekeeping gene).
